# A meta-analysis of clinical effects of microscopic unilateral laminectomy bilateral decompression (ULBD) versus biportal endoscopic ULBD for lumbar canal stenosis

**DOI:** 10.3389/fsurg.2022.1002100

**Published:** 2022-09-23

**Authors:** Guang-Xun Lin, Zhi-Kang Yao, Chen Xin, Jin-Sung Kim, Chien-Min Chen, Bao-Shan Hu

**Affiliations:** ^1^ Department of Orthopedics, The First Affiliated Hospital of Xiamen University, School of Medicine, Xiamen University, Xiamen, China; ^2^ The Third Clinical Medical College, Fujian Medical University, Fuzhou, China; ^3^ Department of Orthopedics, Kaohsiung Veterans General Hospital, Kaohsiung, Taiwan; ^4^ Department of Neurosurgery, Seoul St. Mary’s Hospital, College of Medicine, The Catholic University of Korea, Seoul, South Korea; ^5^ Division of Neurosurgery, Department of Surgery, Changhua Christian Hospital, Changhua, Taiwan; ^6^ College of Nursing and Health Sciences, Dayeh University, Changhua, Taiwan; ^7^ School of Medicine, Kaohsiung Medical University, Kaohsiung, Taiwan

**Keywords:** unilateral biportal endoscopic, biportal endoscopic spinal surgery, lumbar canal stenosis, microscopic surgery, unilateral laminotomy bilateral decompression

## Abstract

**Osbjective:**

Several studies have shown that both microscopic unilateral laminotomy bilateral decompression (ULBD) and unilateral biportal endoscopic (UBE) ULBD are effective for treating lumbar canal stenosis (LCS). However, there are different viewpoints as to which surgical technique is superior. Therefore, this meta-analysis investigated the clinical efficacy and side effects of microscopic ULBD and UBE ULBD for treating LCS.

**Methods:**

To identify relevant studies describing the clinical outcomes and complication rates of microscopic ULBD and UBE ULBD for LCS, several databases were systematically searched in the Internet. The visual analog scale score for back and leg pain and the Oswestry Disability Index were used to assess clinical outcomes. Furthermore, data about perioperative outcomes and complications were documented.

**Results:**

In total, six studies with 450 participants were included in this meta-analysis. The UBE ULBD was found to be superior to microscopic ULBD in terms of efficacy against early postoperative back and leg pain. However, there was no significant difference between the two procedures in terms of final clinical outcomes and complications. In addition, compared with microscopic ULBD, UBE ULBD was associated with a significant reduction in the length of hospital stay and C-reactive protein levels 2 days after surgery.

**Conclusion:**

UBE ULBD and microscopic ULBD for the treatment of LCS were similar in terms of final clinical outcomes and complications. However, UBE ULBD has several advantages over microscopic ULBE, including a shorter hospital stay and faster alleviation of postoperative back and leg pain.

## Introduction

Back pain, radiating pain, claudication, and difficulty walking are the indications of lumbar canal stenosis (LCS) (1–3). This condition is defined as narrowing of the spinal canal that might compress the nerve roots and cause neurological symptoms (2, 4). In most cases, conservative therapy is recommended in the early stages. However, with disease progression, the severity of neurologic symptoms also worsens. This results in a significant loss of functional capability and deterioration in the quality of life and, subsequently, the need for surgical intervention (5, 6).

Surgical intervention primarily aims to relieve symptoms and improve function by decompressing nerve structures. Compared to traditional open decompression surgery, minimally invasive microscopic unilateral laminectomy with bilateral decompression (ULBD) techniques can preserve more intact spinal structures, prevent postoperative instability, and minimize soft tissue injury (7–9), and it has favorable results in the treatment of LCS (10, 11).

Recently, novel endoscopic strategies for treating lumbar spinal stenosis using the unilateral biportal endoscopic (UBE) approach have been gaining increasing attention (12, 13). Via a clear magnified view, free bone and tissue manipulation, safe and efficient nerve decompression can be achieved (14).

Recent studies have found that UBE ULBD and microscopic ULBD are both effective for managing LCS. However, there is no definitive evidence to suggest which surgical approach is more advantageous for the treatment of LCS. Therefore, this systematic review and meta-analysis assessed the clinical outcomes and side effects of UBE ULBD and microscopic ULBD in treating LCS.

## Materials and methods

### UBE surgical procedure

**Figure 1:**
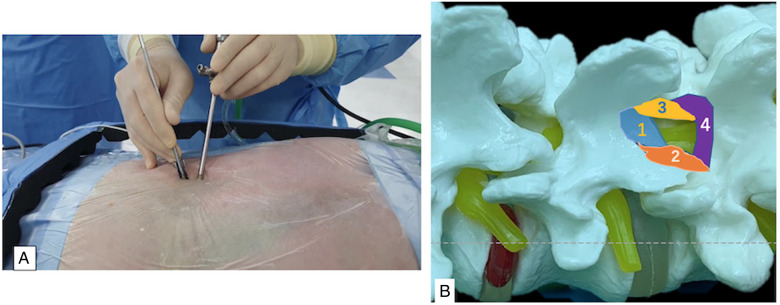
(**A**), intraoperative overview of percutaneous biportal endoscopic approach. (**B**), steps of decompression procedures: 1, spinolaminar junction first, until the origin of ligamentum flavum; 2, ipsilateral laminotomy and medial facetectomy; 3, contralateral sublaminar to lateral recess; 4. upper lamina margin of the lower vertebra.

**Figure 2:**
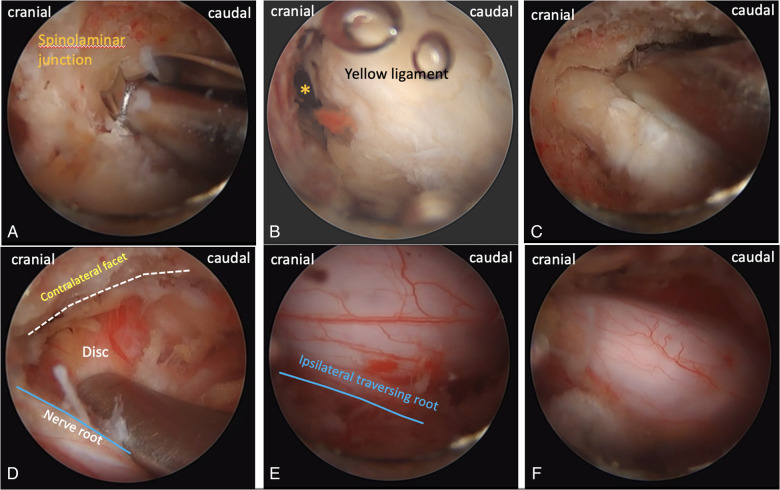
Intraoperative endoscopic images. (**A**), drilling from spinolaminar junction. (**B**), * origin of ligamentum flavum. (**C**), contralateral sublaminar drilling. (**D**), contralateral facet and disc. (**E**), the margin of the ipsilateral nerve root. (**F**), the final view of decompression.

**Figure 3:**
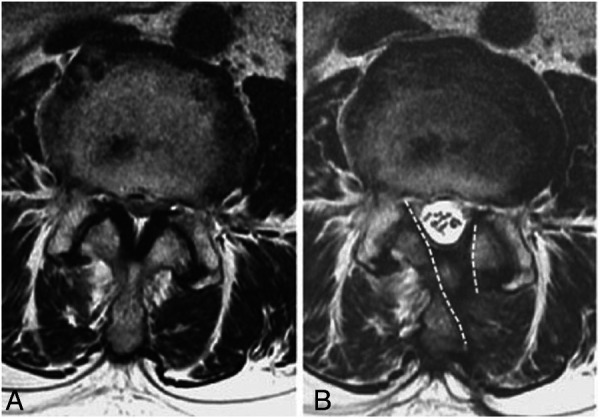
Preservation of facet joint. (**A**), pre-operative and (**B**), post-operative facet joint preservation. Dashed line: the extent of laminotomy.

The skin incisions for the endoscopic portal and working portal were made (Figure 1A). UBE-ULBD requires two incisions, one small incision of approximately 5–6 mm for endoscopic insertion and continuous saline irrigation, and another large incision of approximately 8–10 mm for instrument access and saline outflow. Spinolaminar junction is the initial target area for decompression. Drilling the central portion first instead of the lateral portion could initially avoid excessive facet joint resection. Ipsilateral laminotomy and medial facetectomy were performed. The lower margin of the upper lamina was removed until the origin of ligamentum flavum and underlying epidural fat were exposed. Along with ligamentum flavum, drill the undersurface of the contralateral lamina until the lateral recess is reached. Contralateral facet undercutting has been suggested. Then, resect the upper laminar margin of the lower vertebra. Finally, remove the ligamentum flavum and release the ipsilateral and contralateral traversing nerve root. The procedures were illustrated and described in Figure 1B and Figure 2. In addition, Figure 3 showed that the preservation of facet joint.

### Search strategy

The Preferred Reporting Items for Systematic Reviews and Meta-Analysis criteria were used to conduct a systematic literature review (15). Randomized or nonrandomized controlled trials comparing UBE ULBD and microscopic ULBD for LCS were retrieved. From database inception October 2021, we searched for relevant articles published in the English from PubMed, Embase, Web of Science, and Cochrane Library. The following combinations of keywords were used to achieve the greatest sensitivity in the search strategy: “unilateral biportal endoscopic,” “UBE,” “biportal endoscopic spinal surgery,” “BESS,” “two portal endoscopic spinal surgery,” “microscopic decompression surgery,” “lumbar canal stenosis,” “spinal stenosis,” “unilateral laminectomy bilateral decompression,” and “ULBD.” All reference lists were combed for any fresh research that could be relevant. Two researchers (G.X.L. and B.S.H.) separately examined the titles and abstracts of all identified resources and the entire text of all relevant articles. Conversations and discussions with the third party (C.M.C.) were used to address any disagreements. Ineligibility from the study was documented and reported.

### Selection criteria

The inclusion criteria were as follows: (1) randomized controlled trials (RCTs), or retrospective, or prospective studies, as well as relevant clinical studies/original papers, (2) articles comparing UBE ULBD and microscopic ULBD in humans, (3) studies including patients with LCS managed *via* ULBD using both techniques, (4) studies simultaneously reporting clinical outcomes, such as visual analog scale (VAS) score and/or Oswestry Disability Index (ODI) score, and complications, and (5) studies with a follow-up period of >6 months.

The exclusion criteria were as follows: (1) single-arm studies without comparison groups, (2) studies without relevant data, case reports, review articles, and those unwritten in English, (3) multiple reports from a single center or institution, read the full text and select the largest sample-size study, and (4) duplicate (multiple studies of the same type in the same center or institution).

### Quality assessment

Two reviewers (G.X.L. and B.S.H.) independently assessed the quality of each research included in this meta-analysis. The Modified Jadad scale, which comprises eight items, is used to assess RCTs (16). The scale runs from 0 to 8, with 4–8 denoting good or high quality and 0–3 denoting poor or low quality. For non-RCTs, the quality was evaluated using the Newcastle–Ottawa Scale (NOS) (17). Each study was assessed based on its selection, comparability, and exposure/outcome. Based on this measure, the analyses include studies that received more than five points.

### Data extraction

Two reviewers (G.X.L. and J.S.K.) worked separately to obtain data using standardized data extraction forms. The general characteristics of the studies were as follows: authors, publication year, region, study design, sample size, diagnosis, intervention details, sex, age, and follow-up. The primary outcomes, which comprised preoperative, early (<3 months) postoperative, and final postoperative measurements, were VAS score for back and leg pain and ODI score. The secondary outcomes were perioperative characteristics (average operative time, C-reactive protein [CRP] level, and length of hospital stay) and complications.

### Statistical analysis

The retrieved data were analyzed using Review Manager 5.4 (Cochrane Collaboration, Oxford, the UK). The ×^2^ test was used to determine trial heterogeneity, which was then quantified using the I^2^ statistics. A *P* value of <0.10 was used to assess heterogeneity. The mean differences and 95% confidence intervals (CIs) were used to present continuous data. In comparative studies, dichotomous variables were calculated using odds ratio (OR) or risk ratio. Meanwhile, continuous variables were calculated using weighted mean difference (WMD) or standard mean difference. Significant heterogeneity was defined as *I*^2^ ≥ 50%, and a random-effects model was used for meta-analysis. However, if *I*^2^ < 50%, a fixed effects model was used. A *P* value of <0.05 was considered statistically significant.

## Results

### Selection results and quality evaluation

**Figure 4:**
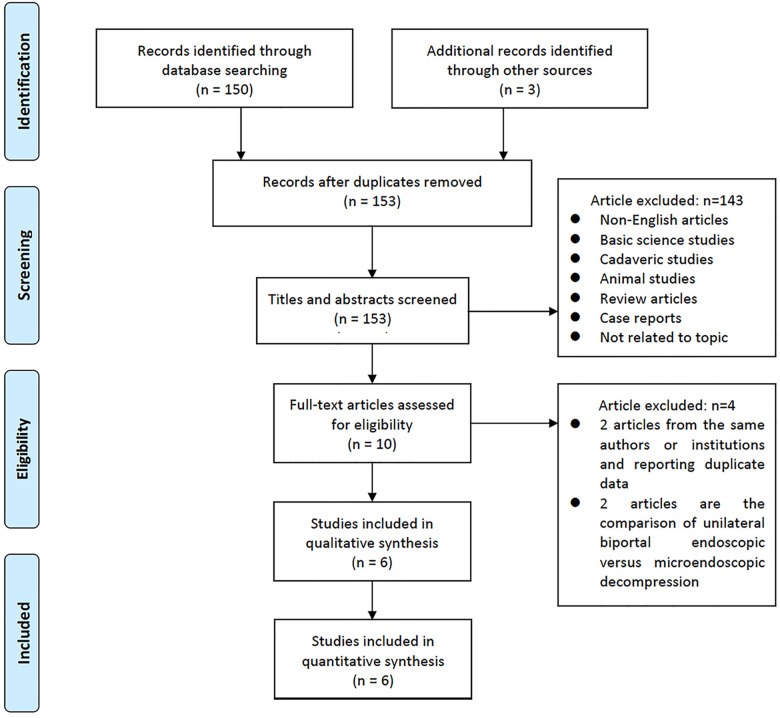
Study selection flow diagram for the meta-analysis.

Initially, 153 studies were identified. In total, 143 studies were eliminated after screening for the titles and abstracts. The remaining 10 studies were thoroughly examined. Finally, six papers matched the criteria, and they were included in this study (18–23). Figure 4 shows the Preferred Reporting Items for Systematic Reviews and Meta-Analysis flowchart, which depict the detailed search strategy.

Two of the studies were RCTs, and four were either prospective (1) or retrospective (3). Based on the Modified Jadad scale and NOS assessment, all studies had moderate-to-high quality (Table 1).

**Table 1:** Quality assessment of the included studies.

A, Modified Jadad Scale for RCTs									
Studies	Was the study described as randomized?	Was the method of randomization appropriate?	Was the study described as blinded?	Was the method of blinding appropriate?	Was there a description of withdrawals and dropouts?	Was there a clear description of the inclusion/exclusion criteria?	Was the method used to assess adverse effects described?	Was the method of statistical analysis described?	Total scores (of 8)
Kang 2019	Yes	Yes	Yes	Unclear	Yes	Yes	Yes	Yes	7
Park 2020	Yes	Yes	Yes	Yes	Yes	Yes	Yes	Yes	8
B, Newcastle-Ottawa Scale for non-RCTs									
Studies	Selection				Comparability	Exposure			Total scores (of 9)
	Is the case definition adequate?	Representativeness of the cases	Selection of Controls	Definition of Controls	Comparability of cases and controls on the basis of the design or analysis	Ascertainment of exposure	Same method of ascertainment for cases and controls	Non-Response rate	
Heo 2018	⋆		⋆	⋆	⋆⋆	⋆	⋆		7
Choi 2019	⋆		⋆	⋆	⋆⋆	⋆	⋆		7
Min 2019	⋆	⋆	⋆	⋆	⋆⋆	⋆	⋆		8
Kim 2020	⋆	⋆	⋆	⋆	⋆⋆	⋆	⋆		8

### Study characteristics

The studies initially included 450 patients with LCS, 226 underwent UBE ULBD and 224 microscopic ULBD. However, three and two patients in the UBE ULBD and microscopic ULBD groups, respectively, were lost to follow-up. Interestingly, all studies were conducted in South Korea. The average ages of the UBE ULBD and microscopic ULBD groups were 52.0 and 51.8 years, respectively. The male: female ratios of the UBE ULBD and microscopic ULBD groups were 128:131 and 138:159, respectively. Table 2 summarizes the basic study characteristics.

**Table 2:** Characteristics of the included studies.

Study	Study design	Country	No. of cases	Diagnosis	Operative level	Age (years)	Sex (M/F)	Follow-up (months)
Heo 2018	Prospective case-control study	South Korea	UBE (46)	All single-level lumbar spinal stenosis	L2-3 (1); L3-4 (8); L4-5 (33); L5-S1 (4)	65.8 ± 8.9	18/28	14.5 ± 2.3
			Microsurgery (42)		L2-3 (2); L3-4 (7); L4-5 (30); L5-S1 (3)	63.5 ± 10.5	16/26	
Choi 2019	Retrospective	South Korea	UBE (35)	Single-level lumbar spinal stenosis (24); multiple levels (11)	N/A	65.4 ± 11.8	14/21	24
			Microsurgery (30)	Single-level lumbar spinal stenosis (15); multiple levels (15)		65.2 ± 12.0	17/13	
Kang 2019	RCT	South Korea	UBE (32)	All single-level lumbar spinal stenosis	L3-4 (4); L4-5 (16); L5-S1 (12)	65.1 ± 8.6	18/14	6
			Microsurgery (30)		L3-4 (5); L4-5 (15); L5-S1 (10)	67.2 ± 9.5	14/16	
Min 2019	Multicenter retrospective case-control study	South Korea	UBE (54)	All single-level lumbar spinal stenosis	L2-3 (1); L3-4 (7); L4-5 (43); L5-S1 (2)	65.71 ± 10.51	27/27	27.2 ± 5.4
			Microsurgery (35)		L2-3 (1); L3-4 (7); L4-5 (24); L5-S1 (3)	66.74 ± 7.96	19/16	31.5 ± 7.3
Kim 2020	Retrospective	South Korea	UBE (30)	All single-level lumbar spinal stenosis	L2-3 (2); L3-4 (8); L4-5 (18); L5-S1 (2)	64.23 ± 5.26	13/17	12
			Microsurgery (30)		L2-3 (4); L3-4 (8); L4-5 (16); L5-S1 (2)	66.20 ± 6.01	12/18	
Park 2020	RCT	South Korea	UBE (32)^a^	All single-level lumbar spinal stenosis	L1-2 (0); L2-3 (2); L3-4 (5); L4-5 (25); L5-S1 (0)	66.20 ± 6.01	13/19	12
			Microsurgery (32)^b^		L1-2 (3); L2-3 (3); L3-4 (7); L4-5 (17); L5-S1 (2)	66.20 ± 6.01	18/14	

^a^ 3 cases lost to follow-up before 12 months.

^b^ 2 cases lost to follow-up before 12 months.

UBE, unilateral biportal endoscopic; RCT, Randomized Controlled Trial.

### Clinical outcomes

In four studies (18, 19, 22, 23), the VAS score for back and leg pain was presented as mean ± standard deviation (*n* = 328 at the preoperative period; *n* = 164 patients who received UBE ULBD and *n* = 164 patients who underwent microscopic ULBD). During the postoperative follow-up, there were 161 patients in the UBE ULBD group and 162 in the microscopic ULBD group.

**Figure 5:**
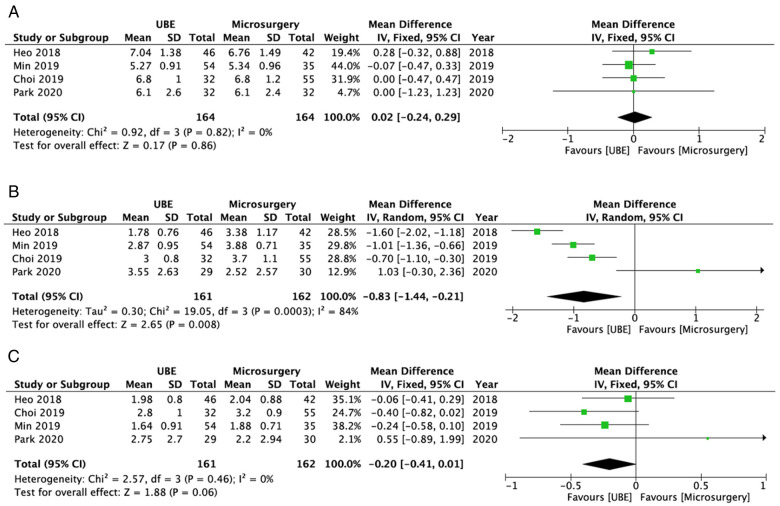
Forest plots for comparison of VAS for back at preoperative (**A**), early (<3 months) postoperative (**B**), and final follow-up (**C**) between UBE ULBD and microscopic ULBD. VAS, visual analog scale; UBE, unilateral biportal endoscopic; ULBD, unilateral laminectomy with bilateral decompression.

The preoperative average VAS score for back pain of the UBE ULBD and microscopic ULBD groups did not significantly differ (WMD: 0.02; 95% CI: −0.24, 0.29; *I*^2^ = 0%; *P* = 0.86; Figure 5A). The early postoperative average VAS score for back pain was significantly higher in the microscopic ULBD group than in the UBE ULBD group (WMD: −0.83; 95% CI: −1.44, −0.21; *I*^2^ = 84%; *P* = 0.008; Figure 5B). There was no significant difference in terms of average VAS score for back pain between two groups during the final follow-up (WMD: −0.20; 95% CI: −0.41, 0.01; *I*^2^ = 0%; *P* = 0.06; Figure 5C).

**Figure 6:**
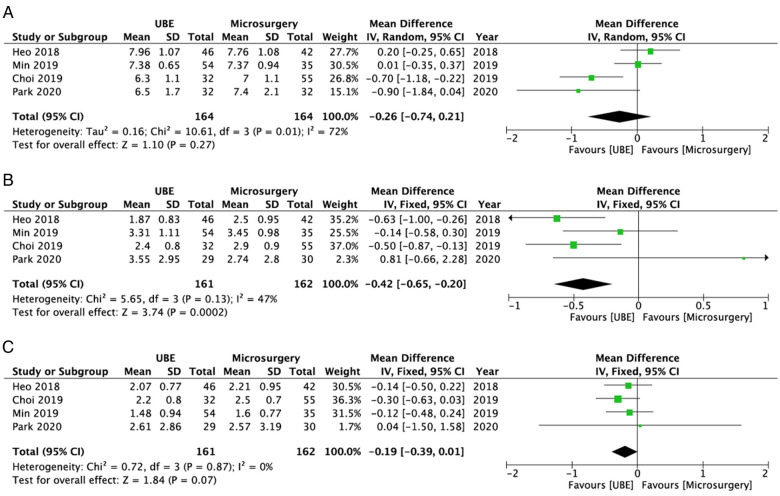
Forest plots for comparison of VAS for leg at preoperative (**A**), early (<3 months) postoperative (**B**), and final follow-up (**C**) between UBE ULBD and microscopic ULBD. VAS, visual analog scale; UBE, unilateral biportal endoscopic; ULBD, unilateral laminectomy with bilateral decompression.

The UBE ULBD and microscopic ULBD groups did not significantly differ in terms of mean preoperative VAS score for leg pain (WMD: −0.26; 95% CI: −0.74, 0.21; *I*^2^ = 72%; *P* = 0.27; Figure 6A). Compared with microscopic decompression, UBE ULBD was associated with a significantly early postoperative leg pain alleviation (WMD: −0.42; 95% CI: −0.65, −0.20; *I*^2^ = 47%; *P* = 0.0002; Figure 6B). The two techniques were similar in terms of the final postoperative VAS score for leg pain (WMD: −0.19; 95% CI: −0.39, 0.01; *I*^2^ = 0%; *P* = 0.07; Figure 6C).

**Figure 7:**
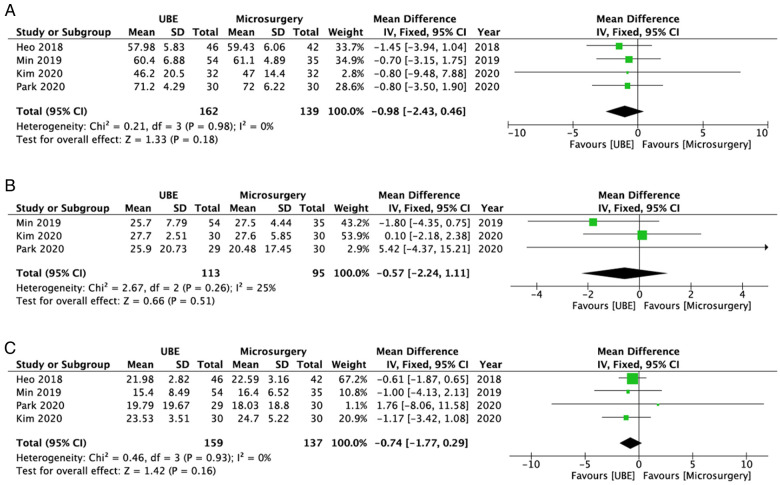
Forest plots for comparison of ODI at preoperative (**A**), early (<3 months) postoperative (**B**), and final follow-up (**C**) between UBE ULBD and microscopic ULBD. ODI, Oswestry Disability Index; UBE, unilateral biportal endoscopic; ULBD, unilateral laminectomy with bilateral decompression.

Four studies (19, 21–23) reported data about ODI scores during the preoperative period (*n* = 162 patients who received UBE ULBD and *n* = 139 patients who underwent microscopic ULBD) and final follow-up (*n* = 159 patients who received UBE ULBD and *n* = 137 patients who underwent microscopic ULBD). Among these studies, only three (21–23) provided data about early postoperative ODI (*n* = 208; 113 patients who received UBE ULBD and 95 patients who underwent microscopic ULBD). There was no statistically significant difference in ODI between the UBE ULBD and microscopic ULBD groups during the preoperative period (WMD: −0.98; 95% CI: −2.43, 0.46; *I*^2^ = 0%; *P* = 0.18; Figure 7A), early postoperative period (WMD: −0.57; 95% CI: −2.24, 1.11; *I*^2^ = 25%; *P* = 0.51; Figure 7B), and final follow-up (WMD: −0.74; 95% CI: −1.77, 0.29; *I*^2^ = 0%; *P* = 0.16; Figure 7C).

**Figure 8:**
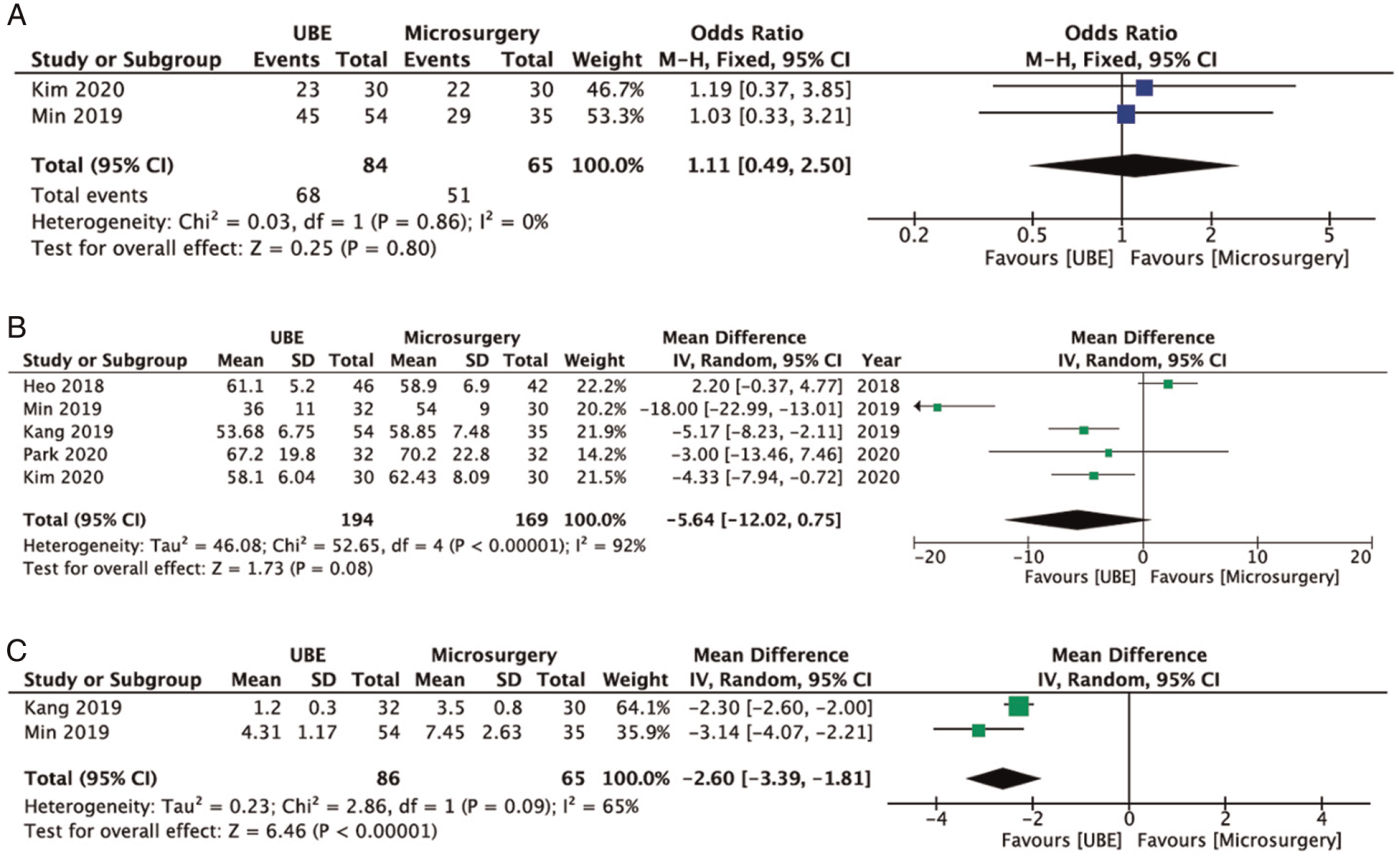
Forest plots comparing satisfaction rates (**A**), operative time (**B**), and length of hospital stay (**C**) between UBE ULBD and microscopic ULBD. UBE, unilateral biportal endoscopic; ULBD, unilateral laminectomy with bilateral decompression.

Two studies (21, 22) compared the satisfaction rate between the UBE ULBD (*n* = 84) and microscopic ULBD (*n* = 65) groups. Results showed no significant difference in terms of satisfaction rate (OR: 1.11; 95% CI: 0.49, 2.50; *I*^2^ = 0%; *P* = 0.80; Figure 8A). In addition, one RCT provided a more comprehensive clinical data in terms of European Quality of Life-5 Dimensions score and painDETECT score. At the 3-, 6-, and 12-month follow-ups, there were no differences in European Quality of Life-5 Dimensions and painDETECT scores between the groups.

### Perioperative measurements

Five studies (*n* = 363 cases; 194 patients who received UBE and 169 who underwent microscopic) provided data about mean operative time (19–23). The two groups were similar in terms of mean operative time (WMD: −5.64; 95% CI: −12.02, 0.75; *I*^2^ = 92%; *P* = 0.08; Figure 8B).

Two studies (*n* = 151; 86 in the UBE ULBD group and 65 in the microscopic ULBD group) provided information about the length of hospital stay (20, 22). The UBE group had a shorter length of hospital stay than the microscopic group (WMD: −2.60; 95% CI: −3.39, −1.81; *I*^2^ = 65%; *P* < 0.00001; Figure 8C).

**Figure 9:**
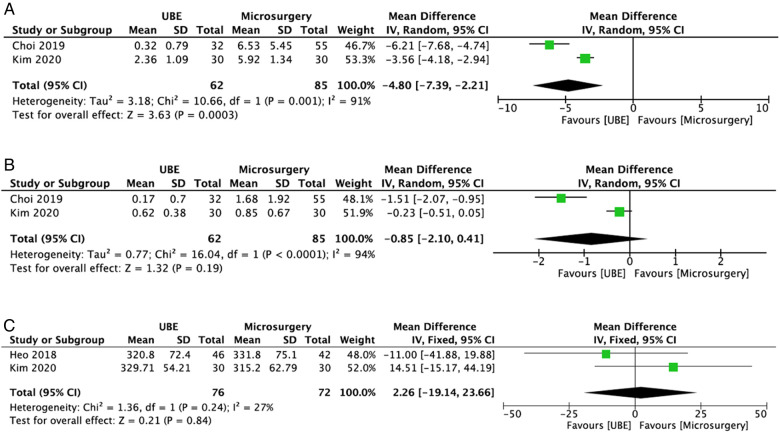
Forest plots comparing CRP level at 2-day after surgery (**A**) and 1-week after surgery (**B**), and postoperative dural expansion (**C**) between UBE ULBD and microscopic ULBD. CRP, C-reactive protein; UBE, unilateral biportal endoscopic; ULBD, unilateral laminectomy with bilateral decompression.

Two studies (18, 21) compared the CRP levels between the UBE ULBD (*n* = 62) and microscopic ULBD (*n* = 85) groups. On the second postoperative day, the serum CRP level of the UBE ULBD group was lower than that of the microscopic ULBD group (WMD: −4.80; 95% CI: −7.39, −2.21; *I*^2^ = 91%; *P* = 0.0003; Figure 9A). However, the serum CRP level at 1 week after surgery did not differ significantly between the two groups (WMD: −0.85; 95% CI: −2.10, 0.41; *I*^2^ = 94%; *P* = 0.19; Figure 9B).

Kim et al. (21) showed that the serum creatine kinase level of the UBE ULBE group (130.87 ± 51.49 mg/dl) was significantly lower than that of the microscopic ULBD groups (331.40 ± 118.09 mg/dl) 2 days after surgery. However, Park et al. (23) revealed that the serum creatine phosphokinase level did not differ significantly between the two groups (151.0 [107.0−216.8] IU/I vs. 111.0 [83.3−230.3] IU/I) 48 h after surgery.

Kang et al. (20) showed that UBE ULBD was associated with a significantly lower volume of drainage compared with microscopic ULBD (25.5 ± 15.8 ml vs. 53.2 ± 32.1 ml, *P* = 0.043). By contrast, Park et al. (23) showed that the Hemovac drain output after UBE ULBD was significantly larger than that after microscopic ULBD (97.5 [70.0−163.0] ml vs. 27.5 [12.6−53.9] ml, *P* < 0.001).

The demand for opioid analgesics decreased after UBE compared with microscopic decompression, according to Park et al. (23) and Kang et al. (20) The time to ambulation after UBE ULBD was faster than that after microscopic ULBD (7.77 ± 3.14 h vs. 16.68 ± 2.96 h, *P* < 0.001) based on the study of Min et al. (22).

### Dural expansion

In terms of radiological outcomes, two studies (19, 21) compared the development of dural expansion between the UBE ULBD (*n* = 76) and microscopic ULBD group (*n* = 72) groups. The incidence rate of postoperative dural expansion was comparable between the UBE and microsurgery groups (WMD: 2.26; 95% CI: −19.14, 23.66; *I*^2^ = 27%; *P* = 0.84; Figure 9C). Furthermore, the dynamic intervertebral angle, percentage of slip, and dynamic slip did not change significantly between the two groups according to the study of Min et al. (22).

### Complications

**Figure 10:**
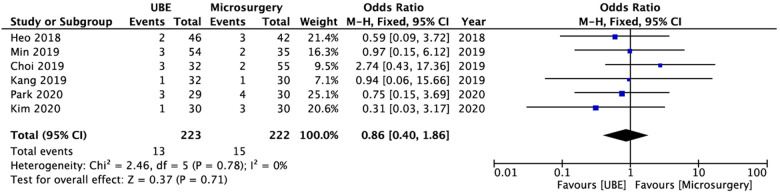
Forest plot for comparions of complications between between UBE ULBD and microscopic ULBD. UBE, unilateral biportal endoscopic; ULBD, unilateral laminectomy with bilateral decompression.

All six studies (*n* = 445; *n* = 223 who received UBE ULBD and *n* = 222 who underwent microscopic ULBD) reported data about complications (18–23). In terms of complications, there were no significant differences between the UBE ULBD and microscopic ULBD groups (OR: 0.86; 95% CI: 0.40, 1.86; *I*^2^ = 0%; *P* = 0.71; Figure 10). Table 3 shows details about complications in the included studies. The incidence rates of complications in the UBE ULBD and microscopic ULBD groups were 5.8% and 6.8%, respectively. The most common complications were dura tear and hematoma.

**Table 3:** Comparison of complications between the two groups.

	UBE (*n* = 223)	Microsurgery (*n* = 222)
Dura tear	7	6
Hematoma	4	5
Cerebrospinal fluid leak	1	2
Root injure	1	0
Infection	0	1
Recurrent pain	0	1
Total	13	15

UBE, unilateral biportal endoscopic.

## Discussion

According to several studies, the outcomes of UBE ULBD and microscopic ULBD for the treatment of LCS are effective and comparable (20, 21, 24). With the high resolution and magnification of the endoscope, the UBE provides a clearer surgical view (25). Simultaneously, the split of viewing and working portals in UBE surgery allows the surgeon to utilize both hands freely, thereby facilitating a smoother instrument handling (18, 21). The surgeon can conduct sufficient contralateral decompression with less posterior facet violation by tilting the working portal. This surgical technique offers the essential advantages of endoscopic surgery, preserving the bilateral facet joints more than microscopic ULBD (26). In addition, continuous pressure saline irrigation maintains the sterility of the surgical area (19). Therefore, UBE ULBD is a feasible alternative to microscopic ULBD.

The current study revealed that both UBE and microscopic ULBD can achieve satisfactory clinical outcomes, and both procedures did not significantly differ in terms of the final follow-up clinical (ODI, VAS score, and satisfaction) and radiological outcomes. Simultaneously, UBE ULBD was found to be superior to microscopic ULBD in the alleviation of back and leg pain (VAS) during the early postoperative period. Furthermore, compared with microscopic ULBD, UBE ULBD can considerably reduce time to ambulation, use of opioid analgesics, and length of hospitalization. These results are attributed to less muscle retraction during UBE since the portals are placed percutaneously, and tissue injury was reduced due to less dissection. In addition, with compared microscopic ULBD, UBE ULBD could significantly reduce trauma due to the preservation of posterior osseous components, such as the lamina and facet (27). These advantages can be indirectly expressed. That is, the CRP and the serum creatine kinase levels 2 days after surgery, which are associated with surgical invasiveness, were significantly lower in UBE ULBD than in microscopic ULBD. However, there are some objections. Despite the fact that the UBE approach involves just a small incision and minimal muscle stripping from the lamina, it also involves muscle division and shaving of the working region, which can elevate the serum creatine phosphokinase level after UBE ULBD (23).

According to the current meta-analysis, the incidence of UBE ULBD and microscopic ULBD complications were 5.8% and 6.8%, respectively. There is no significant difference between the two groups. The most prevalent complications of both groups were dura tear and hematoma. Dura tear is a serious complication. In cases of large-scale dura rupture, we should convert the endoscopic procedure to an open microscopic procedure; small intraoperative durotomies can be sutured with sealant materials (TachoComb or TachoSil), and the patient should be placed on bed rest (19). The most significant step in reducing the incidence of this technical issue is to maintain the operative field clear by preventing epidural hemorrhage. Epidural hematoma can be reduced using a high magnification of the operative field in conjunction with continuous saline irrigation. Technically when we start to remove flavum or performing laminectomy, it was necessary to confirm that there was adequate water flow and bleeding management, particularly on the contralateral side (18). If all efforts fail to stop the bleeding, reducing the diastolic blood pressure to about 100 mmHg might be beneficial in certain circumstances (18). To prevent dural tears, postop hematomas, and other complications and maintain the surgical field clear during UBE, it is essential to mention that the saline flow must be continuous. Therefore, the triangular approach must be appropriate. Using different cannulas or sheaths for the portal designated as a working channel facilitates the exit of the irrigated fluid. In addition, the stagnation of liquid favors an incorrect visualization of the surgical field.

A dedicated pressure pump is recommended in increasing the height of the saline bag or in compressing it to increase the saline pressure (18). Moreover, high-pressure irrigation is not advised since it might increase intracranial pressure and cause delayed postoperative recovery (28). To minimize potential iatrogenic injury, water pressure should be managed between 4.41 cm H_2_O (2.41 mmHg) and 31.00 cm H_2_O (22.83 mmHg) during UBE (29). In addition, the risk factors for epidural hematoma after UBE were preoperative anticoagulant usage, female sex, old age, intraoperative water infusion pump application, and surgery requiring greater bone manipulation (30). In addition, Kang et al. (20) and Park et al. (23) Showed the opposite results of drainage amount after the UBE ULBD and microscopic ULBD. The reason is the irrigation saline during operation inﬁltrated into surrounding muscle and leaked into the drain after surgery. Another reason for this is that bleeding controlled by the water pressure during operation may have drained out postoperatively.

There is also a period of adaptation on the learning curve for UBE ULBD, including extended operative time for decompression owing to a murky surgical field caused by minor bleeds and, recurrent dura rupture caused by a lack of information on the endoscopic vision and surgical anatomy. As previously observed, after roughly 30 instances, its surgical competency is equivalent to that of microscopic ULBD (19). Reassuringly, the endoscope’s field of vision is identical to that of microscopic surgery. As a consequence, experienced spine surgeons can more easily follow the UBE technique. In contrast, inexperienced surgeons need to complete approximately 60 surgical operations and may obtain beneficial results (28).

The current study had some limitations. First, only six articles were included in the study. Among them, four were non-RCTs, and only two were RCTs. Second, the included studies have a small sample size. Third, several important factors could not be investigated due to lack of data. Fourth, considering that these results are based on the capability of the technique in a single region (South Korea), the clinical results of this meta-analysis may be limited because other Western countries may not have the same experience UBE ULBD as spinal endoscopists in South Korea. The impact of the operators' surgical experience on outcomes cannot be overlooked. That is, the outcomes might have been impacted by differences in operating procedures and surgical competence.

## Conclusion

In terms of final clinical outcomes and complications, UBE ULBD and microscopic ULBD for the treatment of LCS were comparable. However, compared to microscopic ULBE, UBE ULBD has several advantages such as shorter hospital stay and faster postoperative relief of back and leg pain. To synthesize high quality data and establish a more reliable recommendation for practice, multicenter RCTs containing more cases should be conducted to compare UBE ULBD with microscopic ULBD.

## Data Availability

The original contributions presented in the study are included in the article/**Supplementary Material**, further inquiries can be directed to the corresponding author/s.
